# Effectiveness of treatments for people living with severe dementia: A systematic review and meta-analysis of randomised controlled clinical trials^☆^

**DOI:** 10.1016/j.arr.2022.101758

**Published:** 2022-12

**Authors:** Elena Profyri, Phuong Leung, Jonathan Huntley, Vasiliki Orgeta

**Affiliations:** University College London, UK

**Keywords:** Severe dementia, Pharmacological treatments, Non-pharmacological treatments, Randomized controlled clinical trials

## Abstract

**Background:**

Dementia is a progressive neurodegenerative syndrome that has no cure. Although a significant proportion of people with dementia progress into the severe stages of the disease, evidence on the clinical effectiveness of treatments for people with severe dementia remains limited.

**Aims:**

To systematically review the effectiveness of pharmacological and non-pharmacological treatments for people living with severe dementia and assess the quality of the evidence.

**Method:**

We searched MEDLINE, EMBASE, PsycINFO, CINAHL and online clinical trial registers up to January 2022, for Randomised Controlled Trials (RCT) in people living with severe dementia. Quality and risk of bias were assessed independently by two authors.

**Results:**

A total of 30 trials met our inclusion criteria of which 14 evaluated the effectiveness of pharmacological treatments, and 16 evaluated a non-pharmacological intervention. *Pharmacological treatments:* Meta-analyses indicated that pharmacological treatments (donepezil: 10 mg, 5 mg; galantamine: 24 mg; memantine: 10 mg) are associated with better outcomes compared to placebo for: severity of symptoms (standardized mean difference (SMD) 0.37, 95% CI 0.26–0.48; 4 studies; moderate-certainty evidence), activities of daily living (SMD 0.15, 95% CI 0.04–0.26; 5 studies; moderate-certainty evidence), and clinical impression of change (Relative Risk (RR) 1.34, 95% CI 1.14–1.57; 4 studies; low-certainty evidence). Pharmacological treatments were also more likely to reduce mortality compared to placebo (RR 0.60, 95% CI 0.40–0.89; 6 studies; low-certainty evidence). *Non-pharmacological treatments:* Five trials were included in the meta-analyses of non-pharmacological interventions (multi-sensory stimulation, needs assessment, and activities-based interventions); results showed that non-pharmacological interventions may reduce neuropsychiatric symptoms of dementia compared to usual care (SMD −0.33, 95% CI −0.59 to −0.06; low certainty evidence).

**Conclusions:**

There is moderate-certainty evidence that pharmacological treatments may decrease disease severity and improve function for people with severe dementia. Non-pharmacological treatments are probably effective in reducing neuropsychiatric symptoms but the quality of evidence remains low. There is an urgent need for high-quality evidence for other outcomes and for developing service-user informed interventions for this under-served group.

## Highlights

• Moderate-certainty evidence shows that pharmacological treatments may decrease disease severity and improve function in severe dementia.
• Non-pharmacological interventions may reduce neuropsychiatric symptoms but evidence remains of low-quality.
• The evidence-base of effectiveness of treatments for people with severe dementia remains small and should be a priority for future research.

## 1 Introduction

Dementia is a progressive neurodegenerative syndrome affecting approximately 50 million people worldwide (Livingston et al., 2020). Although significant progress has been made in the development and evaluation of interventions for people living with mild and moderate dementia, evidence-base of treatments for people living with severe dementia remains substantially limited (Livingston et al., 2017). Prevalence figures estimate that around 12.5 % of people above the age of 60, and 17.3 % of those aged over 85 years are affected by severe dementia (Prince et al., 2013), with both of these numbers expected to rise sharply in the next 25 years (Wittenberg et al., 2020). Despite this large projected increase, and that over 20 % of the population of people with dementia currently live with severe dementia (Farlow, 2005), this group remains under-treated (Edvardsson et al., 2008), is often excluded by research evaluating treatments (Clare et al., 2010), and remains a highly under-served population (Shepherd et al., 2019).

Progression to severe dementia is characterised by a marked loss in cognition and the ability to carry out activities of daily living (ADL), leading to earlier care home admission (Jonsson et al., 2006). Living with severe dementia is associated with the highest community and informal care costs, which double from the milder to the more advanced stages of the disease (Ku et al., 2016). People with severe dementia are also at greater risk of experiencing frequent and severe behavioural and psychological symptoms, which can be very distressing, and constitute the largest contributor of increased costs of care (Lacey et al., 2017).

Current clinical guidelines on the pharmacological management of severe dementia recommend the use of cholinesterase inhibitors in severe Alzheimer’s disease (AD) with more recent recommendations of the addition of memantine to anticholinesterase inhibitors for severe AD (O'Brien et al., 2017, Schmidt et al., 2015). Although several systematic reviews exist reporting on the clinical effectiveness of pharmacological interventions for people with moderate to severe dementia (Birks and Harvey, 2018), most are now out of date (McShane et al., 2019), and neither has focused on effectiveness of pharmacological treatments specifically for people with severe dementia. Therefore, estimates of clinical effectiveness of pharmacological treatments for this under-represented group remain largely unknown.

Although recent clinical guidelines recommend the use of psychological interventions for preventing or reducing neuropsychiatric symptoms in severe dementia (NICE, 2018), a recent Cochrane review identified no such trials in people living with severe dementia (Orgeta et al., 2022). Despite the multiple reviews in the literature examining the effectiveness of non-pharmacological interventions in dementia, most provide only a narrative synthesis, and are focused on people living with moderate to severe dementia (Hui et al., 2021). As a result, effectiveness of these treatments for people with severe dementia remains unknown with no quantitative estimates available. An important aim of the present review therefore was to evaluate the effectiveness of all types of non-pharmacological interventions for this group, which is typically excluded from both psychosocial and psychological clinical trials research (Orgeta et al., 2022).

This systematic review and meta-analysis aimed to evaluate current evidence of the clinical effectiveness of both pharmacological and non-pharmacological treatments for people living with severe dementia in all settings, in order to inform clinical guidelines, and clinical practice. A secondary objective was to rate the quality of the evidence to date and to make recommendations for future research in the area.

## 2 Methods

This systematic review was conducted based on the guidelines of Preferred Reporting Items for Systematic Reviews and Meta-Analyses (Moher et al., 2009), and was registered with PROSPERO (CRD42021193086). The titles, abstracts and full text of articles were screened by two reviewers independently.

### 2.1 Search strategy

We searched relevant terms to severe dementia and randomised controlled trials (RCTs) (see Appendix in the Supplement) up to January 2022 in major health databases (MEDLINE, EMBASE, PsycINFO, CINAHL; the Cochrane Dementia and Cognitive Improvement Group’s Specialized Register and clinical trials registers). We additionally added the terms ‘moderate’ and ‘moderate to severe dementia’ in our search to avoid missing any mixed sample studies that provided separate data for people with severe dementia (see Supplement for details of the search terms). We additionally hand searched reference lists of identified articles to ensure no studies were missed.

### 2.2 Inclusion criteria

We included: a) RCTs of interventions b) in people with severe dementia, with a diagnosis of any type, c) living in any setting (community, nursing homes, hospitals, inpatient settings). Multicomponent trials, and studies reporting on combinations of pharmacological and non-pharmacological interventions were also eligible. Our judgements of definitions of severe dementia were based on cut-off scores of tools used widely in the published literature, which included the Mini Mental State Examination (MMSE) (Folstein et al., 1975), the Global Deterioration Scale (GDS) (Reisberg et al., 1982), the Functional Assessment Staging Tool (FAST) (Reisberg, 1988), and the Clinical Dementia Rating Scale (CDR) (Hughes et al., 1982). Inclusion criteria for severity of dementia were based on the use of a cognitive screening tool, and/or global assessment scales that measured overall function and disease severity.

Studies that did not provide separate data for people with severe dementia were excluded (e.g., studies reporting on a mixed sample or studies reporting data on people with moderate-to-severe dementia). We additionally excluded studies that did not use a screening tool to assess dementia severity, and studies on the effectiveness of palliative care or end of life care interventions. Studies assessing effectiveness of continuation and discontinuation of treatments were also excluded.

### 2.3 Statistical analysis and quality assessment

We used a fixed-effects model to represent overall estimate effects, which assumes that all studies are estimating the same (fixed) treatment effect, and quantified heterogeneity by using the I^2^ statistic. All calculations were conducted using Review Manager (RevMan) 5.2 for Windows (Cochrane Collaboration, Oxford UK; www.cc-ims.net/RevMan). Where data were available, we collected the number of participants for whom the outcome was measured in each group, means and standard deviations (SDs). We used change from baseline scores for all analyses reported and, if necessary, calculated the change scores. We used Cochrane's tool for assessing risk of bias (Higgins et al., 2011), and the GRADE approach to summarize overall certainty of evidence (Guyatt et al., 2011).

## 3 Results

**Fig. 1:**
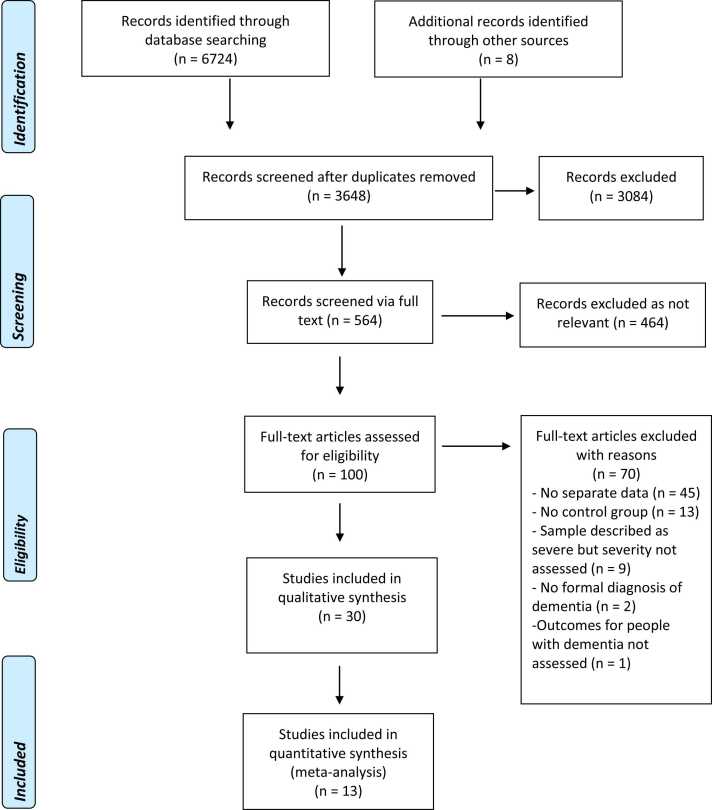
Flowchart of the search and study selection process.

A total of 6724 journal articles were identified by the search (see Fig. 1 for details of the search process), with 8 additional articles identified via hand search. After removing duplicates 3648 articles remained, of which 564 were screened via full text. After removing studies that were not relevant 100 articles were assessed for eligibility. Of these studies, 70 were excluded with reasons (see Appendix in the Supplement) and 30 met inclusion criteria. There were fourteen studies evaluating the clinical effectiveness of pharmacological treatments, of which one tested the effectiveness of a plasma infusion treatment, and sixteen trials of non-pharmacological interventions (see Table 1, Table 2, Table 3 for Characteristics of Included studies). We identified two trials that had multiple treatment groups; for these studies, we combined the two control conditions and then divided the combined group in line with the methods recommended by Cochrane. We were not able to perform any sensitivity analyses due to the small number of studies. We assessed publication bias by producing funnel plots and inspecting them visually for all analyses combining six studies or more (Egger et al., 1997). Due to the small number of studies combined in meta‐analyses, we did not conduct statistical tests for funnel plot asymmetry.

**Table 1:** Characteristics of Included studies of pharmacological treatments (donepezil, galantamine, and memantine) for dementia severity and global function.

**Study**	**Sample and setting**	**Outcomes**	**Treatment**	**Follow-up time points**
Black 2007	n = 343 (F=241; M=102) Mean age: 78.0; Mean MMSE: 7.45 Diagnosis: AD	Primary outcomes 1. SIB 2. CIBIC-Plus Secondary outcomes 3. MMSE 4. ADCS-ADL-sev 5. NPI 6. CBQ 7. RUSP	Donepezil 10 mg/day (n=176) Placebo (n=167)	- 8, 16, and 24 weeks (primary endpoint)
	Inclusion criteria:			
	1. MMSE ≤ 12 2. FAST ≥ 6 3. Modified HIS ≤ 6 4. Carer with contact ≥ 4 hours per day			
	Definition of severe dementia: MMSE ≤ 12 and FAST score ≥ 6			
	Community			
	- 98 centers in USA, Canada, France, UK, & Australia			
Burns 2009 (SERAD study)	n = 407 (F=329; M=78) Mean age: 83.6; Mean MMSE: 8.95 Diagnosis: AD and AD with CVD	Primary outcomes 1. SIB 2. MDS-ADL (7 items) Secondary outcomes 3. Sub items of SIB 4. MDS-ADL (11-items)	Galantamine 24 mg/day (n=207) Placebo (n=200)	- 8, 12, and 26 weeks (primary endpoint)
	Inclusion criteria:			
	1. MMSE 5-12 2. History of cognitive decline 3. CT/MRI scan 3 years before enrolment 4. Sufficient vision & hearing 5. Resident for ≥ 6 months			
	Definition of severe dementia: MMSE 5-12			
	57 Nursing homes			
	- 10 European coun*t*ries			
Feldman 2005 (MSAD study)	n = 145 (F=86; M=59) Mean age: 73; Mean MMSE: 8.94 Diagnosis: AD	Primary outcomes 1. CIBIC-Plus Secondary outcomes 2. sMMSE 3. SIB 4. DAD 5. IADL 6. PSMS 7. NPI	Donepezil 10 mg/day (n = 72) Placebo (n = 73)	- 4, 8, 12, 18, and 24 weeks (primary endpoint)
	Inclusion criteria:			
	1. FAST ≥ 6 2. MMSE 5-12 3. Available carer			
	Definition of severe dementia: sMMSE 5-12			
	Community/assisted living facilities			
	- Canada, Australia, & France			
Hannestad 2021	n = 26 (F=16; M=10) Mean age= 73.5; Mean MMSE: 5.4 Diagnosis: AD Inclusion criteria: 1. Aged 65-90 years 2. MMSE ≤ 10	Primary outcomes 1. MMSE 2. SIB 3. ADCS-ADL-sev 4. NPI	GRF6019 intravenous infusions of 250 mL IV over 5 days (n=18) Placebo (n=8)	- 9 weeks (primary endpoint)
	Definition of severe dementia: MMSE 0 -10			
	Community and 4 nursing homes/ long-term care facilities			
	- United States			
Homma 2008^b^	n = 302 (F=245; M=57) Mean age: 78.2; Mean MMSE: 7.76 Diagnosis: AD	Primary outcomes 1. SIB 2. CIBIC-Plus Secondary outcomes 3. ADCS-ADL-sev 4. BEHAVE-AD	Donepezil 5 mg/day (n = 101) Donepezil 10 mg/day (n =96) Placebo (n = 105)	- 24 weeks (primary endpoint)
	Inclusion criteria:			
	1. Aged ≥ 50 years 2. HIS ≤ 6 3. FAST ≥ 6 4. MMSE 1-12			
	Definition of severe dementia: MMSE 1-12 and FAST score ≥ 6			
	Community			
	- Japan			
Jia 2017	n = 313 (F=266; M=110) Mean age: 70.8; Mean MMSE: 7.3 Diagnosis: AD	Primary outcomes 1. SIB Secondary outcomes 2. CIBIC-Plus 3. MMSE	Donepezil 10 mg/day (n=157) Placebo (n=156)	- 24 weeks (primary endpoint)
	Inclusion criteria:			
	1. Aged 50-90 years 2. SIB 10 – 90 3. MMSE 1-12 4. Carer with contact ≥ 4 hours per day			
	Definition of severe dementia: MMSE 1-12 and SIB scores of 10 – 90			
	Hospitals			
	- China			
Reisberg 2003ª	n = 252 (F=170; M=82) Mean age: 76.1; Mean MMSE: 7.9 Diagnosis: AD	Primary outcomes 1. GCI-C 2. ADCS-ADLsev Secondary outc**o**mes 3. SIB 4. MMSE 5. FAST 6. GDS 7. NPI	Memantine 20mg (n=126) Placebo (n=126)	- 12, and 28 weeks (primary endpoint)
	Inclusion criteria:			
	1. Aged ≥ 50 years 2. GDS 5 or 6 3. MMSE 3-14 4. FAST ≥ 6			
	Definition of severe dementia: MMSE 1-10			
	Community			
	- 32 centers, USA			
Winblad 1999	n = 166 (F=96; M=70) Mean age: 68.4; Mean MMSE: 6.3 Diagnosis: Dementia (not further specified)	Primary outcomes 1. GCI-C 2. BGP Secondary outcomes 3. Ferm’s D-test	Memantine 10mg (n=82) Placebo (n=84)	- 12 weeks (primary endpoint)
	Inclusion criteria:			
	1. Aged 60-80 years 2. GDS 5-7 3. MMSE <10 4. Dementia duration > 12 months			
	Definition of severe dementia: MMSE <10 and GDS score 5-7			
	1 psychiatric hospital and 6 nursing homes			
	- Latvia			
Winblad 2006	n = 248 (F=190; M=58) Mean age: 84.9; Mean MMSE: 6.1 Diagnosis: AD	Primary outcomes 1. SIB 2. ADCS-ADL-sev Secondary outcomes 3. GCI-C 4. MMSE 5. NPI	Donepezil 10 mg/day (n=128) Placebo (n=120)	- 12, and 24 weeks (primary endpoint)
	Inclusion criteria:			
	1. Aged ≥ 50 years 2. Ability to walk alone 3. FAST 5-7c 4. MMSE 1-10			
	Definition of severe dementia: MMSE 1 -10 and FAST score 5-7c			
	50 nursing homes			
	- Sweden			

Note: MMSE: Mini-Mental State Examination; FAST: Functional Assessment Staging; HIS: Hachinski Ischemic Score; SIB: Severe Impairment Battery; CIBIC-Plus: Clinician’s Interview-Based Impression of Change-Plus; ADCS-ADL-sev: Alzheimer's Disease Cooperative Study-Activities of Daily Living inventory modified for Severe Patients; NPI: Neuropsychiatric Inventory; CBQ: Caregiver Burden Questionnaire; RUSP: Resources Utilisation for Severe Alzheimer's Disease; CVD: Cerebrovascular disease; MDS-ADL: Minimum Data Set Activities of Daily Living; sMMSE: Severe Mini-Mental State Examination; DAD: Disability Assessment for Dementia; IADL: Instrumental Activities of Daily Living; PSMS: Physical Self-Maintenance Scale; BEHAVE-AD: Behavioral pathology in Alzheimer's disease; GDS: Global Deterioration Scale; GCI-C: Clinical Global Impression of Change; BGP: Behavioral Rating Scale for Geriatric Patients

ªData in people with severe dementia are reported but are not extractable from the primary paper

^b^Data extracted were based on the safety population as reported in the original paper

**Table 2:** Characteristics of Included studies of pharmacological treatments for people with dementia and neuropsychiatric symptoms.

**Study**	**Sample and setting**	**Outcomes**	**Treatment**	**Follow-up time points**
Deberdt 2008 *combined data from three studies	Study 1; n = 99 Studies 2 & 3; n = 72 Diagnosis: AD, vascular dementia, or mixed dementia	Primary outcome 1. MMSE	*Olanzapine for cognition* Study 1 Olanzapine (5, 10, or 15 mg/d) over 6 weeks (n=80) Placebo (n=19) Studies 2 & 3 Olanzapine (1.0, 2.5, 5.0, or 7.5 mg/d & 2.5–10 mg/d) over 10 weeks (n=52) Placebo (n=20)	- 6-10 weeks (primary endpoint)
	Inclusion criteria:			
	1. MMSE ≤ 25 2. Experiencing neuropsychiatric symptoms			
	Definition of severe dementia: MMSE 0-6			
	Community, nursing homes, or assisted living centres			
	- United States			
De Deyn 2005 **combined data from three studies	Sample across all studies n = 530 Diagnosis: AD, vascular dementia, or mixed dementia	Primary outcomes 1. CMAI 2. BEHAVE-AD	*Risperidone for agitation, aggression, and psychosis* Risperidone (n=333) Placebo (n=201) Study 1 Risperidone (fixed 0.25, 0.5, and 1 mg/d) over 12 weeks Study 2: Risperidone (flexible 0,25 – 2.0 mg oral solution) over 12 weeks Study 3: Risperidone (flexible 0,25 – 1.0 mg oral solution) over 12 weeks	- 4, 8, and 12 weeks (primary endpoint)
	Inclusion criteria:			
	1. Aged ≥ 55 years 2. MMSE ≤ 23 3. Experiencing neuropsychiatric symptoms (measured by BEHAVE-AD or CMAI)			
	Definition of severe dementia: MMSE 0-5			
	Nursing homes			
	- United States, Europe, Australia/New Zealand			
Erdal 2018 (DEP.PAIN.DEM study)	n = 92 Diagnosis: AD	Primary outcomes 1. CSDD 2. MOBID-2	*Analgesic treatment for depression* Active analgesic treatment comprising of paracetamol or buprenorphine for depression over 13 weeks Placebo	- 6, and 13 weeks (primary endpoint)
	Inclusion criteria:			
	1. Aged ≥ 60 years 2. MMSE ≤ 20 3. CSDD ≥ 8			
	Definition of severe dementia: MMSE from 0-10			
	47 Nursing homes			
	- Norway			
Magai 2000	n = 31 (F=31; M=0) Mean age= 89.2 Diagnosis: AD	Primary outcomes 1. CSDD 2. GS 3. CMAI 4. AFBS 5. Facial behaviors	*Sertraline for depression* Sertraline 100 mg over 8 weeks (n=17) Placebo (n=14)	- 8 weeks (primary endpoint)
	Inclusion criteria:			
	1. GDS 6 or 7 2. Probable major or minor depression (based on CSDD cut-offs)			
	Definition of severe dementia: GDS 6 or 7			
	Nursing homes			
	- United States			
Mintzer 2006	n = 119 (demographics for severe group not reported) Diagnosis: AD with or without vascular dementia	Primary outcomes 1. BEHAVE-AD 2. CGI-S	*Risperidone for psychosis* Risperidone 1.0-1.5 mg over 8 weeks (n=57) Placebo (n=62)	- 1, 2, 4, and 8 weeks (primary endpoint)
	Inclusion criteria:			
	1. ≥ 55 years 2. MMSE 5 -23 3. ≥ 2 on any item on BEHAVE-AD			
	Definition of severe dementia: MMSE 5-9			
	Nursing homes			
	- United States			

Note: MMSE: Mini-Mental State Examination; BEHAVE-AD: Behavioral pathology in Alzheimer's disease; CMAI: Cohen-Mansfield Agitation Inventory; CSDD: Cornell Scale for Depression in Dementia; MOBID-2: Mobilization-Observation-Behavior-Intensity-Dementia-2; GDS: Global Deterioration Scale; GS: Gestalt Scale; AFBS: Aversive Behaviour Feeding Scale; CGI-S: Clinical Global Impressions scale-Severity

*Study 1 - Street 2000; Study 2 - De Deyn 2004; Study 3 - Deberdt 2005

** Study 1 - Katz 1999; Study 2 - De Deyn 1999; Study 3 - Brodaty 2003

**Table 3:** Characteristics of Included studies of non-pharmacological treatments.

**Study**	**Sample and setting**	**Outcomes**	**Treatment**	**Follow-up time points**
Baker 2003ª	n = 136 (no further details) Diagnosis: AD, vascular or mixed dementia	Primary outcomes 1. REHAB 2. GIP 3. BMD 4. BRS	Multi-sensory stimulation over 4 weeks (n=65) Control group (simple activities such as quizzes and playing cards) (n=71)	- 4 weeks (primary endpoint)
	Inclusion criteria:			
	1. MMSE 0-17			
	Definition of severe dementia: MMSE 0-9 Day hospitals and psycho-geriatric wards - UK, Netherlands, & Sweden			
Ballard 2002ª	n = 72 (F=43; M=29) Mean age: 78.5 Diagnosis: Dementia (not further specified)	Primary outcomes 1. CMAI Secondary outcomes 2. CMAI sub scores 3. NPI 4. Social withdrawal 5. Engaging in activities	Melissa essential oil over 4 weeks (n=36) Placebo oil (n=36)	- 4 weeks (primary endpoint)
	Inclusion criteria:			
	1. Clinically significant agitation assessed by CMAI 2. Cause severe to moderate problems for staff assessed by NPI 3. CDR of 3			
	Definition of severe dementia: CDR score of 3			
	8 NHS nursing homes			
	- UK			
Ballard 2018 ª (WHELD study)	n = 180 (no further details) Diagnosis: Dementia (not further specified)	Primary outcomes 1. DEMQOL-Proxy Secondary outcomes 2. CMAI 3. NPI-MH 4. Antipsychotic use 5. GDS 6. CDR 7. CSDD 8. CANE 9. QuIS 10. Abbey Pain Scale	Person-centered care training for staff, structured tailored activities, and anti-psychotic review over 8 months (n=77) Treatment as usual (n=103)	- 9 months (primary endpoint)
	Inclusion criteria:			
	1. CDR ≥ 1 operationalised to require a minimum level of cognitive, functional, and neuropsychiatric features			
	Definition of severe dementia: FAST score of 7			
	69 NHS nursing homes			
	- UK			
Clare 2013	n = 65 (F=51; M=14) Mean age: 83.4 Diagnosis: AD, vascular dementia, mixed dementia, unspecified dementia, and Pick’s disease	Primary outcomes 1. QUALID Secondary outcomes 2. PRS 3. GADS 4. BASOLL	Communication skills training and supervision over 8 weeks (n = 32) Treatment as usual (n =33)	- 8 weeks (primary endpoint)
	Inclusion criteria:			
	1. FAST 6 or 7 2. No or limited verbal communication			
	Definition of severe dementia: FAST score of 6 or 7 with limited or no verbal communication			
	8 privately owned care homes			
	- UK			
Hjetland 2021 ª (DEM.LIGHT study)	n = 69 (F=68; M=1) Mean age: 84; Mean MMSE: 4 Diagnosis: AD, vascular dementia, Lewy-body dementia, and unspecified dementia	Primary outcomes 1. SDI 2. Actigraphs	Ambient bright light treatment over 24 weeks (n = 33) Placebo control condition (n =36)	- 8, 16 and 24 weeks (primary endpoint)
	Inclusion criteria:			
	1. Aged ≥ 60 years 2. Sleep and neuropsychiatric disturbances, and severely reduced ADLs			
	Definition of severe dementia: FAST score of 6 or 7 and MMSE scores 10 to 1			
	8 nursing home units			
	- Norway			
Hutson 2014	n = 39 (F=34; M=5) Mean age: 86.6; Mean MMSE: 4.9 Diagnosis: AD, vascular dementia, Lewy body dementia, mixed dementia, and unspecified dementia	Primary outcomes 1. RAID 2. CSDD 3. NPI 4. QoL-AD 5. HCS	Multisensory stimulation, reminiscence, and light physical activity over 8 weeks (n=21) Treatment as usual (n=18)	- 8 weeks (primary endpoint)
	Inclusion criteria:			
	1. MMSE 1-17 2. No serious health problem 3. Functionally able to attend group			
	Definition of severe dementia: MMSE; 80% of the sample is described as having severe dementia			
	4 care homes			
	- UK			
Kovach 2004 ª	n = 78 (F=71; M=7) Mean age: 86.6; Median MMSE: 4.64 Diagnosis: Dementia (not further specified)	Primary outcomes 1. ASD 2. Agitation measured by a visual analogue scale 3. Number of therapeutic activity sessions	Controlling and implementing activity schedules to ensure balance between high and low arousal (n=36) Treatment as usual (n=42)	- post-test not specified (primary endpoint)
	Inclusion criteria:			
	1. MMSE ≤ 15 2. Identified by a nurse as having agitation 3. Resident in the home for the next 4 weeks 4. ≤ 4 hours of sleep during the day 5. FAST 6 or 7			
	Definition of severe dementia: MMSE ≤ 9			
	13 long-term care facilities			
	- United States			
Kovach 2006	n = 114 (F=86; M=28) Mean age: 86.5; Mean MMSE: 7.8 Diagnosis: Dementia (not further specified)	Primary outcomes 1. Discomfort-DAT 2. BEHAVE-AD	Assessment of needs, administering non-pharmacological treatments, analgesics, and consultation with practitioners (n=57) Treatment as usual (n=57)	- 4 weeks (primary endpoint)
	Inclusion criteria:			
	1. MMSE indicative of moderate or severe cognitive impairment controlling for levels of education (MMSE ≤ 23) 2. FAST 6 or 7 3. Resident in the home for the next 4 weeks			
	Definition of severe dementia: MMSE and FAST 6 or 7 95% of this sample has a FAST score 6 or 7			
	14 nursing homes			
	- United Stares			
Liu 2017 ª	n = 128 (F=107; M=21) Mean age: 88.6; Mean MMSE: 3.41 Diagnosis: Dementia (not further specified)	Primary outcomes 1. MQS III 2. C-PAINAD	Pain management over 16 weeks (n=64) Treatment as usual (n=64)	- 8, and 16 weeks (primary endpoint)
	Inclusion criteria:			
	1. Aged > 65 years 2. Advanced dementia 3. Substantial communication impairment 4. ≥ 1 painful condition			
	Definition of severe dementia: Score of > 5 on interRAI HC			
	7 nursing homes			
	- Hong Kong			
Olsen 2016	n = 24 (no further details) Diagnosis: Dementia (not further specified)	Primary outcomes 1. CSDD 2. BARS 3. QUALID	Animal assisted activities over 12 weeks (n=12) Treatment as usual (n=12)	- 12 weeks (primary endpoint), and 6 months
	Inclusion criteria:			
	1. Aged ≥ 65 years 2. MMSE ≤ 25			
	Definition of severe dementia: CDR score of 3 (subsample)			
	3 nursing homes			
	- Norway			
Pieper 2018 ª	n = 288 (F=207; M=81) Mean age: 83.8 91.5% of the sample had a GDS score of 6 or 7 Diagnosis: Dementia (not further specified)	Primary outcomes 1. PACSLAC-D 2. MDS-RAI	Multidisciplinary intervention for pain management over 12 weeks (n=148) Treatment as usual (n=140)	- 3 (primary endpoint), and 6 months
	Inclusion criteria:			
	1. GDS ≥ 5 2. MMSE ≤ 25			
	Definition of severe dementia: GDS score of 6 or 7			
	12 Nursing homes - Netherlands			
Reisberg 2017	n =20 (F=15; M=5) Mean age: 78.9; Mean MMSE: 8 Diagnosis: AD	Primary outcomes 1. CIBIC-Plus 2. ADCS-ADL-sev Secondary outcomes 3. SIB 4. MMSE 5. FAST-DS 6. BEHAVE-AD-FW 7. RMBPC 8. GDS	Comprehensive person-centered management over 28 weeks (n=10) Treatment as usual (n=10)	- 4, 12, and 28 weeks (primary endpoint)
	Inclusion criteria:			
	1. Aged ≥ 50 years 2. Living in the community 3. Available carer 4. GDS 5 or 6 5. FAST ≥ 6a 6. MMSE 3-14 7. Receiving memantine			
	Definition of severe dementia: GDS 6 and FAST score ≥ 6a			
	Community			
	- United States			
Sakamoto 2013	n =39 (F=32; M=7) Diagnosis: AD	Primary outcomes 1. Faces Scale 2. Heart rate 3. Heart rate high frequency 4. BEHAVE-AD	Interactive music therapy over 10 weeks (n=13) Passive music therapy over 10 weeks (n=13) Treatment as usual (n=13)	10 (primary endpoint), and 13 weeks
	Inclusion criteria:			
	1. Aged ≥ 65 years 2. CDR = 3			
	Definition of severe dementia: CDR score of 3			
	Care homes and dementia hospital			
	- Japan			
Sánchez 2016 ª	n =32 (F=25; M=7) Mean age= 85.4 Diagnosis: Dementia (not further specified)	Primary outcomes 1. CMAI 2. NPI 3. CSDD 4. sMMSE 5. BANS-S	Multisensory stimulation over 16 weeks (n=11) One-to-one activity sessions over 16 weeks (n=11) Treatment as usual (n=10)	- 8, 16 (primary endpoint), and 24 weeks
	Inclusion criteria:			
	1. Aged ≥ 65 years 2. GDS 6 or 7			
	Definition of severe dementia: GDS score 6 or 7			
	Specialized dementia centers for older people			
	- Spain			
Stenvall 2012 ª	n = 64 Mean age = 82.1 MMSE = 7.7 Diagnosis: Dementia (not further specified)	Primary outcomes 1. Complications 2. S-COVS 3. Katz ADL Index	Multidisciplinary post-operative intervention of rehabilitation (n=28) Treatment as usual (n=36)	- 4 (primary endpoint), 12 months
	Inclusion criteria:			
	1. Aged ≥ 70 years 2. Femoral neck fracture			
	Definition of severe dementia: MMSE ≤ 9			
	Hospitals			
	- Sweden			
Strøm 2017 ª	n = 63 (no further details) Diagnosis: Dementia (not further specified)	Primary outcomes 1. MMSE 2. HCS	Multi-sensory stimulation over 24 weeks (n=29) Reading group (n=15) Treatment as usual (n=19)	- 12, and 24 weeks (primary endpoint)
	Inclusion criteria:			
	1. Aged ≥ 65 years 2. Moderate to severe cognitive impairment with MMSE 0-20			
	Definition of severe dementia: MMSE score of 0-10			
	6 nursing homes			
	- Ireland			

Note: MMSE: Mini-Mental State Examination; REHAB: Rehabilitation Evaluation Hall and Baker tool; GIP: Behavior Rating Scale for Psychogeriatric Inpatients; BMD: Behaviour and Mood Disturbance Scale; BRS, Behaviour Rating Scale; CMAI: Cohen-Mansfield Agitation Inventory; NPI: Neuropsychiatric Inventory; CDR: Clinical Dementia Rating; FAST: Functional Assessment Staging; NPI-NH: Neuropsychiatric Inventory–Nursing Home Version; GDS: Global Deterioration Scale; CSDD: Cornell Scale for Depression in Dementia; CANE: Camberwell Assessment of Need for the Elderly; QuIS: Quality of Interactions Schedule; QUALID: Quality of Life in Late-stage Dementia scale; PRS: Positive Response Schedule; GADS: Guy’s Advanced Dementia Schedule; BASOLL: Behavioural Assessment Scale of Later Life; SDI: Sleep Disorder Inventory; RAID: Rating Anxiety in Dementia Scale; QoL-AD: Quality of Life-Alzheimer’s Disease Scale; HCS: Holden Communication Scale; ASD: Arousal States in Dementia Scale; Discomfort-DAT: Discomfort Dementia of the Alzheimer’s Type; BEHAVE-AD: Behavioral pathology in Alzheimer's disease; interRAI HC: interRAI-Home Care Assessment; MQS III: Medication Quantification Scale version III; C-PAINAD: Chinese-Pain Assessment in Advanced Dementia; BARS: Brief Agitation Rating Scale; PACSLAC-D: Pain Assessment Checklist for Seniors with Limited Ability to Communicate; MDS-RAI: Minimum Dataset of the Resident Assessment Instrument Pain scale; CIBIC-Plus: Clinician’s Interview-Based Impression of Change-Plus; ADCS-ADL-sev: Alzheimer's Disease Cooperative Study-Activities of Daily Living inventory modified for Severe Patients; SIB: Severe Impairment Battery; FAST-DS: Fast Disability Score; RMBPC: Revised Memory and Behavior Problems Checklist; sMMSE: Severe Mini-Mental State Examination; BANS-S: Bedford Alzheimer Nursing Severity Scale; S-COS: Clinical Outcome Variables; Katz ADL Index: Katz Index of Independence in Activities of Daily Living;

ª Study not included in meta-analysis

### 3.1 Pharmacological treatments for severity of dementia and global function

Five studies evaluated the effectiveness and safety of donepezil versus placebo (Black et al., 2007, Feldman et al., 2005, Homma et al., 2008, Jia et al., 2017, Winblad et al., 2006), two studies effectiveness of memantine (Reisberg et al., 2003, Winblad and Poritis, 1999), and one study the efficacy of galantamine (Burns et al., 2009) (see Table 1). Four of these trials were conducted in community settings, three in nursing homes, and one study in a hospital setting. We were able to conduct meta-analyses by extracting data for eight outcomes in total.

#### 3.1.1 Severity of symptoms

**Fig. 2:**
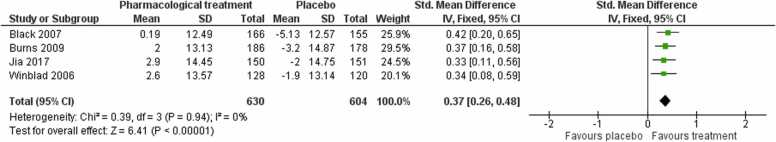
Forest plot of comparison of pharmacological treatments versus placebo for severity of dementia symptoms at post-treatment.

There was moderate‐certainty evidence from four studies that pharmacological treatments may be superior to placebo at improving severity of dementia symptoms at end of treatment (Standardized Mean Difference (SMD) 0.37, 95% Confidence Interval (CI) 0.26–0.48; I² = 0%; 1234 participants; Fig. 2).

#### 3.1.2 Activities of daily living

**Fig. 3:**
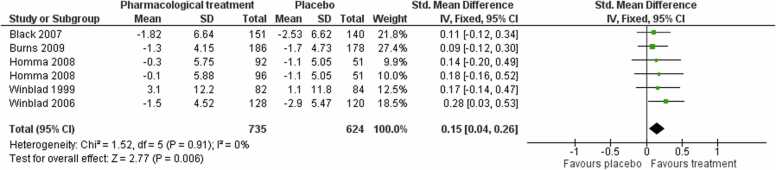
Forest plot of comparison of pharmacological treatments versus placebo for activities of daily living at post-treatment.

Pooling data from five studies showed that pharmacological treatments probably improve patient function compared to placebo at the end of treatment (SMD 0.15, 95 % CI 0.04–0.26; moderate‐certainty evidence; I² = 0 %; one study contributed two independent comparisons; 1359 participants; Fig. 3), representing a small effect.

#### 3.1.3 Global impression of change

Pooling data from four studies (Black et al., 2007, Jia et al., 2017, Winblad et al., 2006, Winblad and Poritis, 1999) showed that pharmacological treatments were probably favoured compared to placebo at improving global impression of change measured as a dichotomous outcome at end of treatment (Risk ratio (RR) 1.34, 95 % CI 1.14–1.57; low-certainty evidence; I² = 0 %; 1009 participants). There was very-low certainty evidence that pharmacological treatments were no different to placebo at end of treatment for global impression of change measured as a continuous outcome (SMD −0.11, 95 % CI −0.25 to 0.02; I² = 34 %; 3 studies (Black et al., 2007, Jia et al., 2017, Winblad et al., 2006); 864 participants).

#### 3.1.4 Cognition

We found low-certainty evidence that pharmacological treatments are probably better than placebo at improving cognition at end of treatment (mean difference (MD) 0.78, 95 % CI 0.33–1.23; I^2^ = 0 %; 3 studies (Black et al., 2007, Jia et al., 2017, Winblad et al., 2006); 832 participants).

#### 3.1.5 Neuropsychiatric symptoms

We pooled four studies to assess the effectiveness of pharmacological treatments on neuropsychiatric symptoms, of which one contributed two independent comparisons. There was low‐certainty evidence that pharmacological treatments may not differ from placebo in their effect on neuropsychiatric symptoms at the end of treatment (SMD −0.06, 95 % CI −0.19 to 0.06; I² = 38 %; 4 studies (Black et al., 2007, Homma et al., 2008, Winblad et al., 2006, Winblad and Poritis, 1999); 1001 participants).

#### 3.1.6 Adverse events

The meta-analyses of the total number of participants experiencing at least one adverse event showed differences in favour of placebo at end of treatment (RR 1.09, 95 % CI 1.03–1.15; I² = 37 %; moderate-certainty evidence; 7 studies, 1924 participants; see Appendix in the Supplement), and no evidence of publication bias (see Appendix in the Supplement).

#### 3.1.7 Serious adverse events

We pooled data from seven studies to examine differences between pharmacological treatments and placebo on the total number of participants experiencing a serious adverse event at end of treatment; there were no differences between the two groups (RR 0.83, 95 % CI 0.67–1.03; I² = 0 %; low-certainty evidence; 7 studies, 1924 participants; see Appendix in the Supplement), and no evidence of publication bias (see Appendix in the Supplement).

#### 3.1.8 Mortality

**Fig. 4:**
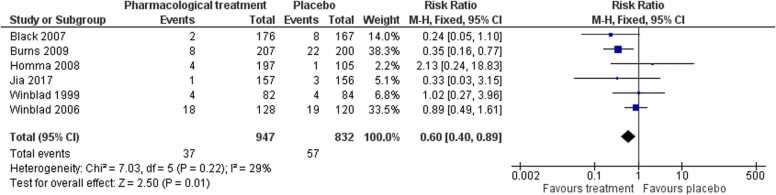
Forest plot of comparison of pharmacological treatments versus placebo for number of deaths at post-treatment.

We found low-certainty evidence that for number of deaths pharmacological treatments were favoured in comparison to placebo at end of treatment (RR 0.60, 95 % CI 0.40–0.89; I² = 29 %; 6 studies, 1779 participants; Fig. 4). There was no evidence of publication bias (see Appendix in the Supplement). We were not able to extract data for the Reisberg et al. (2003) study as these were not available from the primary paper; the authors reported that analyses favoured treatment for all outcomes tested (data not reported). Hannestad et al. (2021) examined the safety, tolerability, and preliminary efficacy of GRF6019 in people with severe dementia living in both the community and nursing home care; overall results indicated that GRF6019 was safe, with good feasibility and tolerability.

### 3.2 Pharmacological treatments for people living with severe dementia and neuropsychiatric symptoms

Five trials tested the effectiveness of pharmacological treatments for people living with severe dementia and neuropsychiatric symptoms. Four studies were conducted in nursing homes, with one study recruiting participants from both community and long-term care settings. Two studies examined the effectiveness of risperidone (De Deyn et al., 2005, Mintzer et al., 2006), and one study (Deberdt et al., 2008) the effectiveness of olanzapine on cognition. Magai et al. (2000) assessed the clinical effectiveness of sertraline in patients with severe AD and depression, and Erdal et al. (2018) an active analgesic treatment versus placebo in people with severe AD and clinically significant symptoms of depression. Due to the small number of studies, we were not able to conduct any meta-analyses, and therefore discuss findings narratively.

#### 3.2.1 Neuropsychiatric symptoms

The study by De Deyn et al. (2005) reported significant differences favouring risperidone at end of treatment; for both agitation (mean change from baseline −13.1 for the risperidone group vs −4.9 for the placebo group; p < 0.001), and overall neuropsychiatric symptoms (mean change from baseline −6.0 for the risperidone group vs −3.1 for the placebo group; p < 0.001). Mintzer et al. (2006) found no differences between groups on psychosis (estimated group difference of risperidone minus placebo at endpoint was −1.2 95% CI: −2.5 to 0.1), but a significant effect favouring treatment on global impression of change (*χ*² _[1]_ = 5.11, p = 0.024).

#### 3.2.2 Cognition

In the study by Deberdt et al. (2008) olanzapine was no different to placebo at end of treatment for cognition in people with severe dementia and neuropsychiatric symptoms (p = 0.78).

#### 3.2.3 Depression

Magai et al. (2000) reported no differences between sertraline versus placebo at post-treatment on both depression scores (sertraline mean at endpoint 3.53 vs 4.43 for placebo), and clinical response at end of treatment (47 % showed ≥ 50 % improvement for sertraline vs 36 % for placebo). Similarly in the study by Erdal et al. (2018) active analgesic treatment comprised of paracetamol or buprenorphine transdermal system was no different to placebo at post-treatment (mean change on depression scores −0.66 in the treatment group vs −3.30 in the placebo group).

### 3.3 Non-pharmacological treatments

A total of sixteen trials evaluated non-pharmacological interventions versus usual care; 13 studies were conducted in nursing homes, with the remaining three trials conducted in day hospitals, general hospitals, or people living with severe dementia at home. There were four trials evaluating multi-sensory stimulation (Baker et al., 2003, Hutson et al., 2014, Sánchez et al., 2016, Strøm et al., 2017); four studies evaluating person-centered care (Ballard et al., 2018, Clare et al., 2013, Kovach et al., 2006, Reisberg et al., 2017); three trials assessing effectiveness of activities-based interventions (Kovach et al., 2004, Olsen et al., 2016, Sakamoto et al., 2013), and two studies evaluating pain management (Liu and Lai, 2017, Pieper et al., 2018). The remaining trials evaluated bright light treatment (Hjetland et al., 2021), aromatherapy (Ballard et al., 2002), and hip fracture rehabilitation (Stenvall et al., 2012). We were able to extract data for two outcomes.

#### 3.3.1 Neuropsychiatric symptoms

**Fig. 5:**
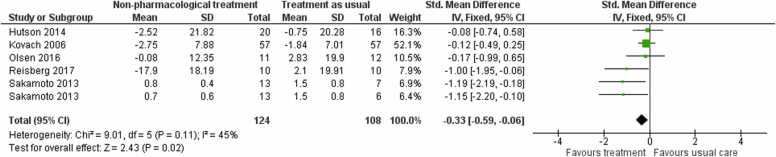
Forest plot of comparison of non-pharmacological treatments versus treatment as usual for neuropsychiatric symptoms at post-treatment.

There was low-certainty evidence that nonpharmacological interventions may reduce neuropsychiatric symptoms at end of treatment (SMD −0.33, 95 % CI −0.59 to −0.06; I² = 45 %; 5 studies; of which one contributed two independent comparisons; 232 participants; Fig. 5).

#### 3.3.2 Quality of life

There was very-low certainty evidence that non-pharmacological interventions may not differ from treatment as usual for patient quality of life at end of treatment (SMD 0.31, 95 % CI ‐0.10–0.71; I² = 0 %; 3 studies (Clare et al., 2013, Hutson et al., 2014, Olsen et al., 2016); 95 participants).

#### 3.3.3 Studies not included in the meta-analyses

##### 3.3.3.1 Multi-sensory stimulation

In the study by Baker et al. (2003) multi-sensory stimulation was associated with lower apathy scores compared to control at end of treatment (mean improvement of −0.4 points for the treatment group vs 0.6 points for the control group). Sánchez et al. (2016) evaluated the effectiveness of multi-sensory stimulation versus one-to-one activity sessions versus a control intervention; multi-sensory stimulation was associated with lower agitation compared to control (p < 0.001; η^2^ =0.30), and greater improvement on severity of symptoms compared to both control and activity sessions (p < 0.001; η^2^ =0.33). Strøm et al. (2017) assessed the effect of multi-sensory stimulation versus usual care versus reading sessions; this study found that those randomised in multi-sensory stimulation had higher communication scores compared to those randomised in the reading sessions (p = 0.044).

##### 3.3.3.2 Person-centered care interventions

Ballard et al. (2018) evaluated the effectiveness of a person-centered care intervention incorporating antipsychotic review for people living with both moderate and severe dementia in nursing homes; although scores favoured the intervention in reducing neuropsychiatric symptoms for people with severe dementia, results were not significant (p = 0.05), and there was no effect on quality of life (p = 0.97).

##### 3.3.3.3 Activities-based interventions

Kovach et al. (2004) compared an activities-based intervention to usual care; the study reported lower agitation scores favouring the intervention group with no interaction effect on whether the person had moderate or severe dementia (p = 0.488).

##### 3.3.3.4 Pain management

Liu and Lai (2017) evaluated pain management for people with severe dementia living in nursing homes versus usual care; although no differences were observed on the use of pain medications, there was a significant reduction on observational pain scores favouring treatment (p < 0.001). Similarly, in the study by Pieper et al. (2018), pain management decreased overall observed pain but not estimated pain compared to usual care (B = −1.21 points 95 % −2.35 to −0.06; p = 0.020).

##### 3.3.3.5 Other non-pharmacological interventions

Hjetland et al. (2021) tested the effectiveness of bright light treatment for people living with severe dementia in nursing homes; sleep was significantly improved in the intervention group compared to control (p < 0.05). In the study by Ballard et al. (2002) aromatherapy reduced overall levels of agitation for people receiving the intervention compared to those receiving placebo (p < 0.0001). Stenvall et al. (2012) examined the effects of a multidisciplinary hip fracture rehabilitation intervention; those randomised to receive the intervention had less post-operative complications such as falls (p = 0.005), and were more likely to regain overall premorbid ADL levels (p = 0.027).

### 3.4 Quality of evidence

None of the studies meeting our inclusion criteria were classified as having low risk of bias in all domains of risk assessment. We considered 9 studies to be at unclear risk of bias, and two studies at high risk, for the domain of random sequence generation. Most studies were judged to be at unclear risk of bias for the allocation concealment domain, due to insufficient information provided, and all but two studies had unclear risk in the domain of performance bias, given that personnel and/or participants were not blinded. For the domain of detection bias we assessed eight studies as having unclear risk, and four studies as having high risk in this domain. Sixteen studies were judged to be at unclear risk in the domain of incomplete outcome data, and in eight studies there was evidence of selective reporting indicative of unclear risk in this domain. Other potential biases were identified only in one study. Detailed ratings for each individual study and across all included studies can be seen in Appendix in the Supplement. Given the small number of studies, we tested for publication bias only for three outcomes.

## 4 Discussion

### 4.1 Summary of findings

To our knowledge, this is the first high-quality systematic review evaluating the clinical effectiveness of treatments for people with severe dementia. Despite several decades of research, our review finds that systematic evaluation of treatments for people with severe dementia remains largely under-developed, with very few large-scale randomised controlled trials in the area. Nevertheless, our review found 30 studies meeting our inclusion criteria indicating that research in this area is growing. Key findings of our review are that moderate-certainty evidence shows that pharmacological treatment (donepezil, memantine, and galantamine) has benefits for people with severe dementia by improving severity of symptoms and activities of daily living. There was also moderate-certainty evidence that pharmacological treatments were more likely to be associated with adverse effects. Results consistent with benefits in terms of improvement of severity of symptoms and a reduction in functional decline in this group are important as they may translate to reduced costs of care (Lacey et al., 2017), delays in care home admission, and reductions in caregiver burden (Ku et al., 2016).

Despite evidence indicating that pharmacological treatments may potentially benefit people with severe dementia on several other outcomes such as global impression of change, cognition, and mortality, certainty of evidence for these outcomes remains low. We are also very uncertain about the effectiveness of pharmacological treatments in reducing neuropsychiatric symptoms in people with severe dementia, which is generally based on very-low certainty evidence. In addition, most of the studies tested effectiveness of donepezil 10 mg/day, so our knowledge of the specific effects of galantamine and memantine for this group, or effect interactions of dosage of treatments remains limited.

A further important limitation in terms of the completeness and applicability of the evidence is the lack of long-term data of effectiveness of pharmacological treatments beyond 26 weeks, and very limited data reporting on patient quality of life, and caregiver outcomes. Future long-term effectiveness trials therefore of the different pharmacological treatments would be important in order to establish whether any benefits persist beyond 6 months. These studies will also be very informative in terms of the effect of incidence of serious adverse events of these treatments and their long-term safety, and whether effect sizes achieve minimum clinically important differences (Andrews et al., 2019). Nevertheless, our findings do support current clinical guidelines recommending continuation of pharmacological treatments for people at more advanced stages of AD (O'Brien et al., 2017, Schmidt et al., 2015). We also found a small number of studies evaluating the effectiveness of pharmacological treatments for people living with severe dementia and neuropsychiatric symptoms. Due however to the small evidence base we are unable to make any conclusions about these treatments.

An important contribution of our review is that it is the first to systematically examine the clinical effectiveness of non-pharmacological treatments for people with severe dementia. We found sixteen studies evaluating the effectiveness of non-pharmacological interventions, which included mostly interventions aimed at multi-sensory stimulation, supporting person-centered care, and activities-based interventions. Our meta-analyses showed that interventions focusing on person-centered care and activities may also reduce neuropsychiatric symptoms compared to usual care, although these results were based on low-certainty evidence. Additionally, the variation between studies in terms of the nature of the interventions, makes the interpretation of these effects less straightforward. Despite these limitations, however, these results compare favourably with minimal or no benefits of pharmacological treatments and the potential for harm from these treatments. Our review additionally identified two trials evaluating the effectiveness of pain management, and although preliminary, both of these studies suggested that pain management may provide important clinical benefits in terms of reducing observational pain in severe dementia (Liu and Lai, 2017, Pieper et al., 2018).

Although finding several studies testing effectiveness of non-pharmacological interventions across many different countries is encouraging, most studies to date tend to be feasibility and acceptability studies as opposed to large-scale clinical effectiveness trials. Although we did identify one large-scale clinical effectiveness trial (Ballard et al., 2018), this study included people with both moderate and severe dementia, and results indicated that the benefits reported for people with moderate dementia did not extend to people with severe dementia. An important gap therefore to be addressed by future research is the development and evaluation of interventions that directly address needs of people with severe dementia, and those contributing to their care. Similarly to pharmacological treatments data on long-term effectiveness of interventions was lacking, and there were limited data on effects of these interventions on carers. Contrary to pharmacological trials however most of the non-pharmacological interventions were conducted in long-term care settings, therefore results may not be applicable to the increasing number of people living with severe dementia at home (Wittenberg et al., 2020).

### 4.2 Implications

The evidence to date indicates that further small trials reporting on the effectiveness of pharmacological treatments would not add to the current evidence base, and that future trials should test the efficacy of these treatments long-term as well as investigating their cost-effectiveness across care settings. Evidence of improvements in severity of symptoms and activities of daily living suggest that pharmacological management of people with severe dementia should be closely monitored as it may prevent further disease progression and delay care home admission. An important finding of our review is the observation that many of the studies meeting our inclusion criteria did not follow Consolidated Standards of Reporting Trials, and for a large number of trials data were not extractable. Addressing this methodological issue will be key for future development in this area. There was also evidence of selective reporting which may introduce bias, and for many outcomes such as global impression of change, cognition, quality of life, and neuropsychiatric symptoms evidence remains of low or very-low certainty.

An important finding of our review is that pharmacological treatments were associated with a small effect in reducing risk of mortality for people with severe dementia. This is an important and original finding highlighting a potential survival advantage for people with severe dementia being prescribed pharmacological treatments, however this conclusion remains uncertain due to low-certainty evidence. Similarly, in regards to tolerability although most trials monitored adverse events, reporting of serious adverse events was not detailed, and the long-term safety of pharmacological treatments remains unknown.

An important observation across all studies meeting our inclusion criteria was that people with severe dementia had limited exposure to medication and access to non-pharmacological treatments, indicating that this group remains largely under-treated (Black et al., 2007). We also found that large-scale trials based on well-defined non-pharmacological interventions were generally missing with many studies including multimodal approaches which combined a variety of treatments, with limited input in terms of lived experiences. Initiatives aimed at harmonising and standardising interventions in the area would be important for pooling data in future meta‐analyses. It will be important for future work in the area to develop dementia care interventions that have been developed alongside people with dementia, family carers, and professionals involved in their care. There is also no evidence base on interventions in other types of dementia such as Parkinson's disease dementia, dementia with Lewy bodies, and frontotemporal dementia.

### 4.3 Limitations

Despite the originality of our findings our review has several limitations. While we employed a systematic approach in identifying studies, we may have still missed trials reporting on outcomes for people with severe dementia. Not all studies used the same ‘definition’ of severe dementia, and although heterogeneity was low in most of our analyses, it is likely that the population differed across studies. Selection bias may have also influenced our results whereby healthier patients with severe dementia may be recruited in these trials. Further longitudinal observational studies are needed to investigate the effects of selection bias in recruiting people living with severe dementia across settings.

Although we extracted data on several outcomes, we could be moderately certain only for the effects of pharmacological treatments on severity of dementia, activities of daily living, and incidence of adverse events. For the remaining analyses, evidence was of low or very-low certainty. Most of the studies included had unclear risk of bias in several domains, and intervention effects were measured at end of treatment, so we are unable to comment on the long-term effectiveness of these treatments. We were also not able to extract data on adverse events considered to be related to treatment as this information was missing from the majority of trials.

## 5 Conclusion

Our review provides the first comprehensive summary estimate of the clinical effectiveness of both pharmacological and non-pharmacological interventions for people with severe dementia. Our findings highlight an important gap in dementia care research for people with severe dementia. Although our results are consistent with clinical guidelines, they nevertheless show a lack of high-quality research in the area and highlight an urgent need to develop evidence-based interventions supporting and improving care for people with advanced dementia.

To conclude, our review finds moderate-certainty evidence that pharmacological treatments may improve severity of symptoms and activities of daily living at end of treatment for people with severe dementia. There is also evidence that non-pharmacological interventions may reduce neuropsychiatric symptoms, however this finding is less certain. It is likely that integrated care where a combination of both pharmacological and psychosocial care is being provided, may result in the greatest clinical benefits for this under-served and under-represented vulnerable population.

## Conflict of interest

The authors have no conflicts of interest to report.

## Data Availability

Data will be made available on request. The data that support the findings of this study are available on request from the corresponding author (VO); please email v.orgeta@ucl.ac.uk.
