# Supplementary material for: Effectiveness of treatments for people living with severe dementia: A systematic review and meta-analysis of randomised controlled clinical trials
Source: Ageing Res Rev. 2022 Dec;82:101758. doi: 10.1016/j.arr.2022.101758 (PMC10580243; doi:10.1016/j.arr.2022.101758)
Supplement: Supplementary file 1 — Supplementary material [file mmc1.docx]

Figure 1 Search strategy of the review

1. severe dement*
2. exp severe dement/
3. exp late-stage dementia/
4. moderate dement*
5. exp moderate dement/
6. severe Alzheimer*
7. advanced Alzheimer*
8. late-stage Alzheimer*
9. moderate to severe Alzheimer*
10. exp moderate Alzheimer/
11. exp severe AD/
12. exp late stage AD/
13. exp moderate AD/
14. exp advanced AD/
15. exp late-stage AD/
16. moderate to severe AD/
17. moderate AD/
18. 1 or 2 or 3 or 4 or 5 or 6 or 7 or 8 or 9 or 10 or 11 or 12 or 13 or 14 or 15 or 16 or 17
19. randomized controlled trial.pt.
20. controlled clinical trial.pt.
21. randomized.ab.
22. randomised controlled trial.tw.
23. random*.ab.
24. randomly.ab.
25. trial.ab.
26. 19 or 20 or 21 or 22 or 23 or 24 or 24
27. 18 and 26

Table 1 Excluded studies

| Study | Reason for Exclusion |
| --- | --- |
| 1. Alili 2020 | RCT of personal and tailored care versus treatment as usual for people with moderate and severe dementia living in nursing homes; no separate data |
| 1. Ancoli-Israel 2003 | RCT investigating three forms of light therapy for agitation in people with severe dementia living in nursing homes; no control group |
| 1. Baker 2001 | RCT of multisensory stimulation versus activity sessions in people with moderate to severe dementia living at home (MMSE scores 0-17; Mean MMSE: 10.96); no control group; no separate data |
| 1. Ballard 2018 | RCT of pimavanserin versus placebo for psychosis for people with AD living in nursing homes (MMSE < 6 and MMSE ≥ 6); no separate data |
| 1. Bautmans 2008 | RCT of cervical spine mobilisation versus socialising visits for swallowing in people with dementia and dysphagia living in nursing homes (MMSE < 24; Median MMSE: 9); no separate data |
| 1. Bürge 2017 | RCT of physical exercise versus treatment as usual for activities of daily living in people with moderate to severe dementia in psychiatric wards (CDR ≥ 2; only 33% of the sample has a CDR of 3); no separate data |
| 1. Burns 2009 | RCT of bright light therapy versus usual care for people with dementia living in nursing homes; severity not assessed |
| 1. Camberg 1999 | RCT of simulated presence versus placebo audio versus usual care in people with dementia living in nursing homes (Mean MMSE: 5.1); no separate data |
| 1. Cantarella 2018 | RCT of doll therapy versus control in people living with severe dementia in nursing homes; severity not assessed |
| 1. Chibnall 2005 | RCT of acetaminophen versus placebo for behavior and psychotropic medication use in people with moderate to severe dementia living in nursing homes (FAST stages 5 and 6); no separate data |
| 1. Collier 2010 | RCT of multisensory intervention versus usual care for people with moderate to severe dementia living in nursing homes (MMSE ≤ 14); no separate data |
| 1. Davison 2016 | RCT of a personalised multimedia device versus usual care for agitation and mood in people with mild to severe dementia living in nursing homes (Mean MMSE: 16.2); no separate data |
| 1. Dowling 2005 | RCT of bright light therapy on rest activity disruption versus control in people living with AD in care homes (MMSE range: 0-23; Mean MMSE: 7); no separate data |
| 1. Farlow 2010 | RCT of high (23mg) versus standard dose (10mg) donepezil in people with moderate to severe dementia living at home (MMSE range: 0-20); no control group; no separate data |
| 1. Farlow 2013 | RCT of 13.3mg rivastigmine versus 4.6mg in people with severe AD living in the community (MMSE range: 3–12; Mean MMSE: 8.8); no control group |
| 1. Farlow 2019 | RCT of bryostatin (20mg versus 40mg) versus placebo for cognition for people with moderately severe to severe AD (MMSE-2 range: 4-15); no separate data |
| 1. Farokhnia 2014 | RCT of *Crocus sativus* L. versus memantine in people with moderate to severe AD living at home (MMSE range: 8–14; Mean MMSE: 11.7); no control group; no separate data |
| 1. Feldman 2001 | RCT of donepezil versus placebo for people with moderate to severe AD living at home (sMMSE range: 5 – 17; FAST ≤ 6; Mean sMMSE: 11.8); no separate data |
| 1. Fisman 1981 | RCT of 2-dimethylaminoethanol versus placebo in people with moderately severe or severe AD in inpatient units; severity not assessed; no separate data |
| 1. Fleischhacker 1986 | RCT of memantine versus placebo for people with AD; sample described as severe; severity not assessed |
| 1. Fossey 2006 | RCT of enhanced psychosocial care versus usual care for antipsychotic use in nursing home residents with moderate to severe dementia (only 58% has a CDR score of 3); no separate data |
| 1. Friedman 1991 | RCT of a walking program versus control for communication in people with moderate to severe AD living in nursing homes (MMSE <19); no separate data |
| 1. Fu 2013 | RCT of aromatherapy with or without massage versus placebo for people with mild to severe dementia living in nursing homes (only 47% of the population has a MMSE score ≤ 9); no separate data |
| 1. Grossberg 2013 | RCT of memantine versus placebo in people with moderate to severe AD taking cholinesterase inhibitors living in the community (MMSE range: 3-14; Mean MMSE: 10.7); no separate data |
| 1. Halek 2020 | RCT comparing two dementia-specific case conference interventions in people living with severe dementia in nursing homes: no control group |
| 1. Hall 2005 | RCT of estrogen versus placebo for people living with advanced dementia and aggression in nursing homes (MSSE range: 0-19); no separate data |
| 1. Han 2017 | RCT comparing donepezil 23mg versus 10mg in people living with moderate and severe dementia in the community (Mean MMSE: 12.4); no control group; no separate data |
| 1. Hanson 2011 | RCT of decision making for feeding options versus usual care for people living with advanced dementia in nursing homes; outcomes for people with dementia not assessed |
| 1. Herrmann 2007 | RCT of valproate versus placebo for agitation and aggression in people living with moderate to severe AD in nursing homes (MMSE <15; Mean MMSE: 4.5); no separate data |
| 1. Herrmann 2013 | RCT of memantine versus placebo for agitation and aggression in people living with moderate to severe AD in the community (MMSE range: 0-15; Mean MMSE: 11.85); no separate data |
| 1. Homma 2016 | RCT of sustained versus immediate release donepezil in people living with severe AD in the community (MMSE range: 1-12); no control group |
| 1. Husebo 2014 | RCT of pain treatment versus treatment as usual on mood in people with moderate or severe dementia living in nursing homes (FAST >4; Mean MMSE: 7.9); no separate data |
| 1. Iwasaki 2004 | RCT of herbal medicine versus placebo for cognition and physical function in people with mild to severe dementia living in nursing homes (MMSE range: 0-25; Mean MMSE: 15.1); no separate data |
| 1. Jablonski 2018 | RCT of managing oral hygiene versus control for people with moderate to severe dementia living in nursing homes (MMSE ≤ 18; Mean MMSE: 6.98); no separate data |
| 1. Jones 2007 | RCT of once-daily versus twice-daily memantine in people with moderate to severe AD living in the community (MMSE ≤ 18; Mean MMSE: 12.1); no control group; no separate data |
| 1. Kim 2016 | RCT of physical exercise in people with moderate to severe AD living in nursing homes (MMSE ≤ 20; Mean MMSE: 14.8); no control group; no separate data |
| 1. Kyomen 1999 | RCT of estrogen therapy versus placebo for aggressive behavior in people with moderate-to-severe dementia living in long-term care (Mean MMSE: 4.71); severity not assessed; no separate data |
| 1. Kuske 2009 | RCT of a staff intervention versus relaxation versus usual care for people with mild to severe dementia living in nursing homes; no separate data |
| 1. Livingston 2019 | RCT of multidisciplinary staff training versus usual care for agitation for people with mild to severe dementia living in nursing homes (only 43% of the sample has a CDR of 3); no separate data |
| 1. Magai 2002 | RCT of staff training versus control for mood in people living with moderate to severe cognitive impairment in nursing homes (Mean MMSE: 3.4); no formal diagnosis of dementia |
| 1. Maseda 2018 | RCT of multi-sensory stimulation versus individualized music in people living with severe dementia in nursing homes (GDS 6 or 7): no control group |
| 1. McCallion 1999 | RCT of staff communication skills versus control in people with moderate and severe dementia living in nursing homes (MMSE <18; Mean MMSE: 5.6); no formal diagnosis of dementia; no separate data |
| 1. Molloy 1991 | RCT of tetrahydroaminoacridine versus placebo on cognition, function, and behavior in people with moderate to severe AD living in the community (Mean sMMSE: 16.7; GDS stages 3-6); no separate data |
| 1. Moore 2010 | RCT of physical activity versus usual care for apathy, agitation, and dietary intake in people with mild to severe dementia living in nursing homes (MMSE <25; Mean MMSE: 12.2); no separate data |
| 1. Moyle 2013 | RCT cross-over trial of a companion robots intervention versus reading groups for quality of life in people with moderate to severe de­mentia living in residential care (Mean MMSE: 7.4); no separate data |
| 1. Moyle 2014 | RCT of foot massage versus quiet response for agitation and mood in people with moderate to severe dementia living in long-term care (MMSE < 18); no separate data |
| 1. Narme 2014 | RCT of a music intervention versus cooking activities in people living with moderate to severe dementia in nursing homes (MMSE ≤ 20, Mean MMSE: 10.2); no separate data |
| 1. O’ Connor 2013 | RCT of lavender oil versus control for agitation in people with mild to severe dementia living in nursing homes (CDR ≥1; Mean MMSE: 6.1); no separate data |
| 1. Ohman 2016 | RCT of physical exercise versus usual care for function in people with mild to severe AD living in the community (CDR 2 or 3; Mean: 15.7); no separate data |
| 1. Olazaran 2013 | RCT of brain stimulation versus control in people with moderate to severe AD and gait dysfunction living in nursing homes (only 62% of the sample has severe dementia; sMMSE: 7.8); no separate data |
| 1. Olin 2001 | RCT of carbamazepine versus placebo for people living with dementia in the community (Mean MMSE: 6.0); severity not assessed |
| 1. Pomeroy 1993 | RCT of physiotherapy versus usual care for mobility in people with severe dementia living in long-term care; severity not assessed |
| 1. Raglio 2010 | RCT of music therapy versus control on behavioral and psychological symptoms for people with moderate to severe dementia living in nursing homes (CDR >2; MMSE ≤18; Mean MMSE: 8.3); no separate data |
| 1. Raglio 2015 | RCT of active music therapy versus individualized listening versus standard care for behavioral and psychological symptoms for people with moderate to severe dementia living in nursing homes (MMSE <18; Mean MMSE: 11.1); no separate data |
| 1. Remington 2009 | RCT of a vitamin formulation versus placebo for people with moderate to severe AD living in nursing homes (Mean MMSE: 11.9); no separate data |
| 1. Ridder 2013 | RCT of individual music therapy versus standard care for agitation in people with moderate to severe dementia living in nursing homes (Mean MMSE: 7.5); no separate data |
| 1. Roach 2011 | RCT of activity exercises versus walking versus control in people with mild to severe AD living in long-term care (Mean MMSE: 9.1); no separate data |
| 1. Rolland 2007 | RCT of exercise versus routine medical care for ADLs in people with mild to severe AD living in nursing homes (MMSE <25; Mean MMSE 8.8); no separate data |
| 1. Rostad 2018 | RCT of a pain management intervention versus usual care for people living with severe dementia in nursing homes; no formal diagnosis of dementia; severity not assessed |
| 1. Sommer 2009 | RCT of oxcarbazepine versus placebo for agitation and aggression in people with moderate to severe dementia living in nursing homes (MMSE range: 0–20; Mean MMSE: 5.8); no separate data |
| 1. Staal 2007 | RCT of multi-sensory behavior versus control for agitation in people with moderate to severe dementia living in inpatient units (Mean MMSE: 15.5); no separate data |
| 1. Summers 1986 | RCT of oral tetrahydroaminoacridine versus placebo for global function in people with moderate to severe AD living in inpatient settings; severity not assessed; no separate data |
| 1. Surr 2020 | RCT of dementia care mapping versus usual care for agitation in people with mild to severe dementia living in nursing homes (only 74% has a FAST of 6 or 7); no separate data |
| 1. Tariot 2004 | RCT of memantine for cognition versus placebo in people with moderate to severe AD living in the community already receiving donepezil (MMSE range: 5-14; Mean MMSE: 10.5; FAST scores 4 -6c); no separate data |
| 1. Tariot 2012 | RCT of stable donepezil versus stable increase in people with moderate to severe AD living at home (MMSE range: 0-20; Mean MMSE 13.8); no control group; no separate data; extension of the Farlow 2010 study |
| 1. van Dyck 2007 | RCT of memantine versus placebo in people with moderate to severe AD living in the community (Mean range: 5-14; Mean MMSE: 10.1); no separate data |
| 1. Venturelli 2011 | RCT of a walking program versus control for cognition and ADLs in people with moderate to severe AD living in nursing homes; (MMSE range: 5-15; Mean MMSE: 12.5); no separate data |
| 1. Wenborn 2013 | RCT of activity provision versus usual care in people with dementia living in nursing homes (Mean MMSE: 5.6); no separate data |
| 1. Williams 2008 | RCT of exercise versus walking for depression in people with moderate to severe AD living in nursing homes (GDS ≥ 3; MMSE range: 0-21; Mean MMSE 7.3); no control group; no separate data |
| 1. Zemlan 1996 | RCT of velnacrine versus placebo for cognition in people with mild to severe AD living in the community (MMSE ≥ 9; Mean MMSE: 17.25); no separate data |

Note: RCT: Randomised controlled trial; MMSE: Mini Mental State Examination; AD: Alzheimer’s disease; CDR; Clinical Dementia Rating; FAST: Functional Assessment Staging Tool; sMMSE: Standardised Mini Mental State Examination; GDS: Global Deterioration Scale; ADLs: Activities of daily living.


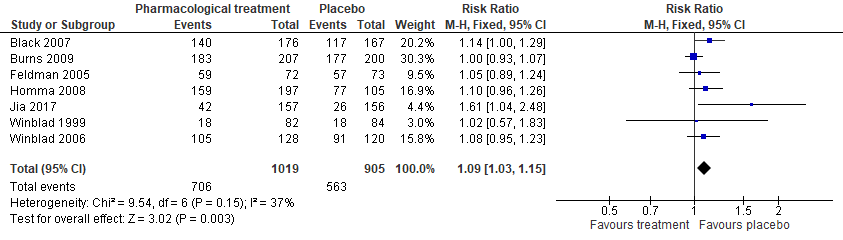


Figure 2. Forest plot of comparison of pharmacological treatments versus placebo for number of any adverse event at post-treatment


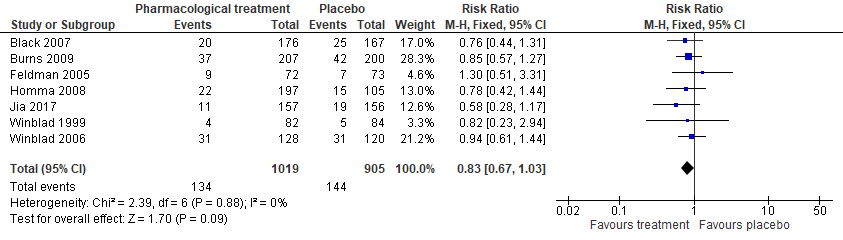


Figure 3. Forest plot of comparison of pharmacological treatments versus placebo for number of serious adverse events at post-treatment


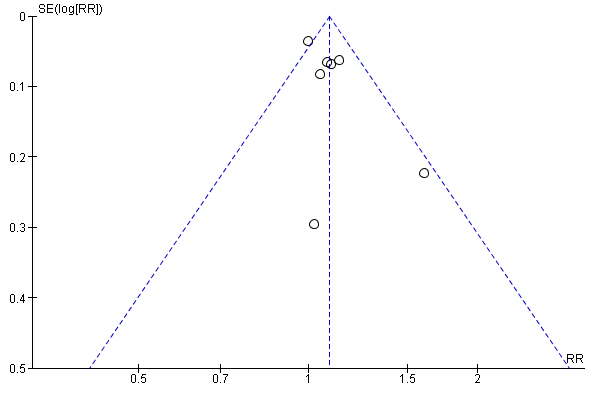


Figure 4. Funnel plot of comparison of pharmacological treatments versus placebo for any adverse event at post-treatment


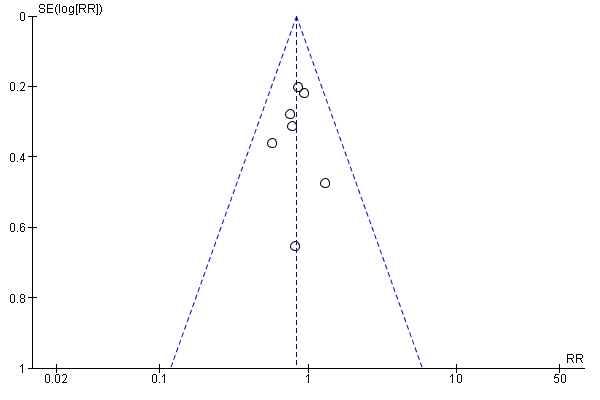


Figure 5. Funnel plot of comparison of pharmacological treatments versus placebo for serious adverse events at post-treatment


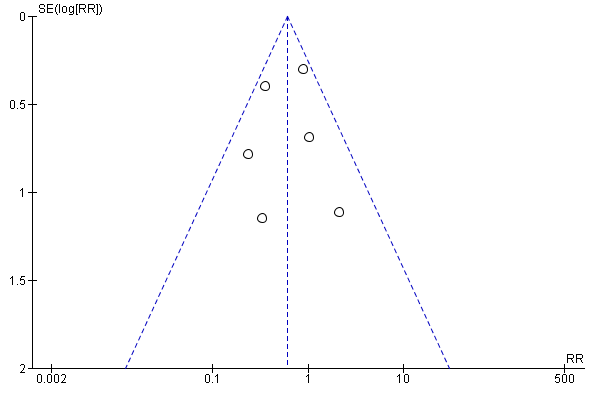


Figure 6. Funnel plot of comparison of pharmacological treatments versus placebo for number of deaths at post-treatment


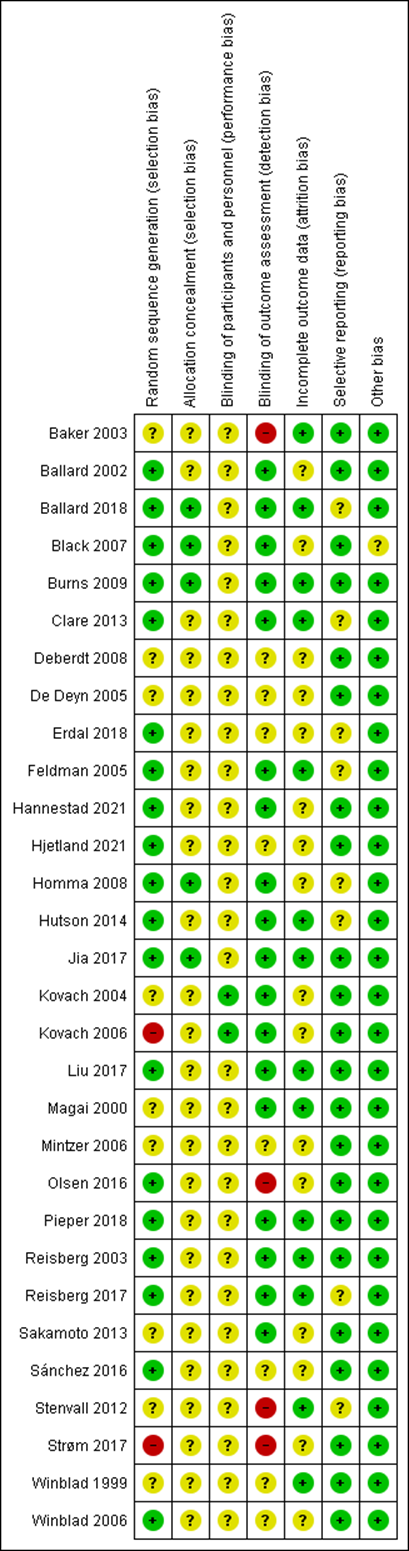


Figure 7. Risk of bias summary: review authors' judgements about each risk of bias item for each included study.


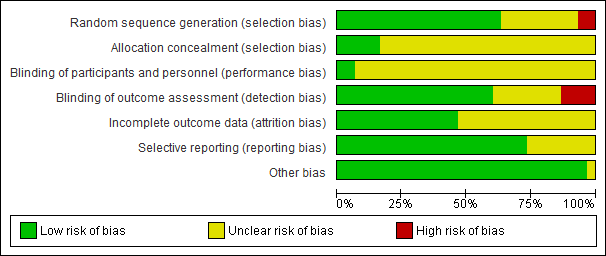


Figure 8. Risk of bias graph: review authors' judgements about each risk of bias item presented as percentages across all included studies.
